# Challenges faced by nurses in implementing aseptic techniques at the surgical wards of the Bamenda Regional Hospital, Cameroon

**DOI:** 10.11604/pamj.2019.33.105.16851

**Published:** 2019-06-12

**Authors:** Tabe Armstrong Tambe, Ngwayu Claude Nkfusai, Frankline Sanyuy Nsai, Samuel Nambile Cumber

**Affiliations:** 1 Department of Public Health, School of Health Sciences, Catholic University of Central Africa, Yaounde, Cameroon; 2 Department of Microbiology and Parasitology, Faculty of Science, University of Buea, Buea, Cameroon; 3 Cameroon Baptist Convention Health Services (CBCHS), Yaounde, Cameroon; 4 Department of Public Health and Hygiene, University of Buea, Buea, Cameroon; 5 Faculty of Health Sciences, University of the Free State, Bloemfontein, South Africa; 6 School of Health Systems and Public Health, Faculty of Health Sciences, University of Pretoria Private Bag X323, Gezina, Pretoria, South Africa; 7 Section for Epidemiology and Social Medicine, Department of Public Health, Institute of Medicine (EPSO), The Sahlgrenska Academy at University of Gothenburg, Gothenburg, Sweden

**Keywords:** Aseptic technique, nurse, hospital acquired infections

## To the editors of the Pan African Medical Journal

Aseptic technique is the use of practices and procedures such as hand hygiene, non-touch techniques, appropriate aseptic field, sterilized equipments and cleaning existing key parts to minimize the presence of disease causing pathogens [1, 2]. While asepsis applies to both medical and surgical procedures which is the absence of potential pathogenic micro-organisms [3, 4]. Compliance with these techniques for infection control is important for the safety of patients and personnel’s as this will reduce nosocomial infections in the units and resulting in patients’ shorter stay in the hospital thus a cost reduction in medical aids unlike when there are infections which will results in increased intuitional cost due to a longer stay in hospital admissions [5]. The goal of aseptic technique is to reach asepsis and each healthcare setting has its own principles and guidelines in achieving asepsis [6]. Preventing surgical site contamination requires the efforts of all in involved in care of the patient to use their theoretical knowledge and experience in aseptic practices to provide patients with optimal care resulting in positive outcomes [7]. In Cameroon there is inadequate data regarding nurse’s knowledge, practices and the barriers they faced in the implementation of aseptic techniques thus giving the investigators the motivation to carry out this investigation. A cross-sectional study was carried out through the administration of a structured questionnaire to healthcare providers (nurses) of the Bamenda Regional Hospital. The study took place at the surgical unit of the Bamenda Regional Hospital, Cameroon. Our study population were nurses working at the surgical unit. All 20 nurses who constantly came in contact with patients with septic wounds for dressing working at the surgical unit were given informed consent and after approval to participate in the study were administered questionnaires. All 20 nurses who constantly came in contact with patients with septic wounds for dressing working at the surgical unit were included in the study. The instrument used for data collection was a well-structured questionnaire designed according to the objectives. The structured questionnaire had two major sections; demographics section and section that investigated the knowledge of the nurses on antiseptic techniques.

**Figure 1:**
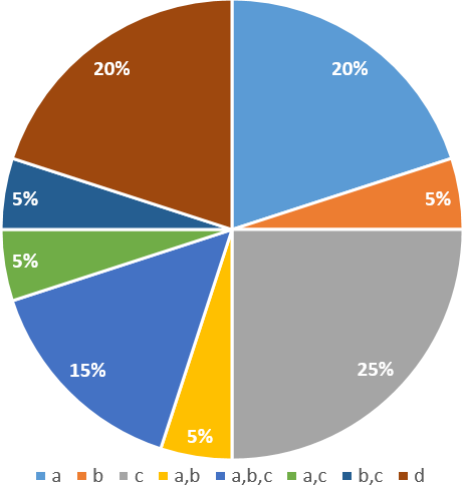
prevention of infections in the surgical ward

Ethical approval was obtained from the Institutional Research Ethics Committee for Human Health at the School of Health Sciences of the Catholic University of Central Africa. Authorization was soughed from the delegate of public health of the North West Region and the director of the Bamenda Regional Hospital to conduct research in his institution. Questionnaires that had initially been tested and validated were administered to the participants. The statistical package Epi Info 7 was used to enter data in this study. SPSS version 20.0, statistical software was used in data cleaning, management and analysis. A descriptive analysis on the cases was done. A total of 20 nurses were used for the studies. Of the nurses, 50% were holders Bachelor of Nursing Science (BNSc), 30% State registered nursing (SRN) and 15% Higher national diploma (HND). Fifty-Five percent were female and 45% male. It was observed that 35% of the nurses had worked for one year or less, while 40% of them had work for 1-5 years and 5-10% of them had work for more than five years. Ninety-five percent of the nurses were christian while 5% of them were muslim. Sixty percent of the nurses were married, 35% single and 5% widow. It was observed that, 85% of nurses face problems of wound infection while 15% do not face any problem. Eighty percent (80%) of the nurses admitted that aseptic techniques are just averagely maintained while 20% strictly follow it. Fifty-five percent of the participants indicated that less than 10 wounds get infected, 25% said 10 to 20 wounds get infected while 20% said more than 20 wounds get infected per year than 20 wounds get infected per year. More than half (75%) of nurses know the causes of infection while 25% don’t identify the causes of infection (Table 1). Of the 75% who identified the causes of infections, 25% noted infections are due to poor aseptic techniques, 20% due to negligence while 5% is due to financial constraints of clients few nurses (45%) use sterile gloves to prevent infection, while 20% use all of the above to prevent the infection and 35% varies (Figure 1). Demographic data showed that female nurses make up 55% of the active population age group of 20-40 years (90%) while 45% were male nurses. Fifty percent of the nurses are state registered nurses where 65% of them have had at least 1 year of working experience. Ninety-five percent of nurses are Christians with 60% married. Considering the level of experience of the nurses, it can be said that the nurses here are knowledgeable and have carried out many wound dressing procedures.

**Table 1:** socio-demographic information

Variable		Frequency/number	Percentage (%)
Age Range (years)	15-20	15	29.4
	21-25	18	35.2
	26-30	12	23.4
	31-35	3	5.88
	36-40	3	5.88
	Total	51	100%
Marital status	Married	33	64.7
	Single	6	11.7
	Separated	6	11.7
	Divorce	3	5.5
	Widow	3	5.5
	Total	51	100%
Duration of pregnancy (Month)	0-3	6	11.7
	4-6	15	39.4
	7-9	30	58.2
	Total	51	100%
Number of Pregnancies	1^st^ pregnancy	24	47.1
	2^nd^ pregnancy	12	23
	More than 2	15	29
	Total	51	100
Are there problems of wound infection	Yes	17	85
	No	3	15
	Total	20	100%
Evaluation of strict usage of aseptic technique in the ward	Very strictly	4	20
	Averagely	16	80
	Poorly	0	0
	Total	20	100%
Number of wounds that get infected per year	Less than 10	11	55
	10-20	5	25
	More than 20	4	20
	Total	20	100%
Are the causes of infection identified	Yes	15	75%
	No	5	25%
	Total	20	100%

Only 20% of the nurses follow aseptic techniques strictly while 80% follow it averagely some of the reasons being less supply of equipment’s and financial constraints of patients. This omission of not maintaining this procedure strictly can be the reason why 85% of nurses face the problem of wounds getting infected where as if it is maintained strictly, the problem of infections could be reduced. This is in line with work done by [8] who states that if corrective changes are implemented in about one-quarter of 1% wound dressings, hospitals could prevent over 100,000 infections each year [9].

The main barriers to the non-application of these aseptic techniques and the major challenges the nurses face is as a result of patient’s financial constraints and inadequate supply of dressing materials. This is in line with a study that was carried out at the Bamenda Health district which revealed that inadequate or absence of basic equipment’s and supplies and also insufficient human resource are the main barriers of nurses implementing aseptic techniques [10]. The study however contradicts the results from our study by stating that the non-appliance of these aseptic techniques by nurses results from a great lack of knowledge and also negligence in implementing this technique by nurses.

## Competing interests

The authors declare no competing interests.
